# Supplementary material for: Non-linear impacts of local fiscal expenditure on farmers’ income: A SHAP-informed machine-learning analysis
Source: PLoS One. 2026 Mar 16;21(3):e0340008. doi: 10.1371/journal.pone.0340008 (PMC12991217; doi:10.1371/journal.pone.0340008)
Supplement: Attachment — Submitted filename: Response to Reviewers.docx [file pone.0340008.s007.docx]

Manuscript ID: PONE-D-25-30509

Title: Non-linear Impacts of Local Fiscal Expenditure on Farmers’ Income: A SHAP-Informed Machine-Learning Analysis

Dear Editor and Reviewers,

We thank you very much for your careful reading of our manuscript and for the constructive comments and suggestions. We have carefully revised the paper in accordance with the recommendations. Below, we provide a point-by-point response, indicating how each comment has been addressed in the revised manuscript. All changes are highlighted in the Revised Manuscript with Track Changes.

**Responses to Editorial Requests**

**Comment 1 (Formatting & File naming)**: Please ensure that your manuscript meets PLOS ONE's style requirements.

**Response:** We reformatted the manuscript according to the PLOS ONE template, adjusted section headings and file naming, and ensured figures and tables follow journal style.

**Comment 2 (ORCID):** Please ensure the corresponding author has a validated ORCID.

**Response:** The corresponding author has validated her ORCID iD in the Editorial Manager system. The ORCID iD is 0009-0000-3824-5829.

**Comment 3 (Funding):** The funding information does not match between “Funding Information” and “Financial Disclosure.”

**Response:** We have corrected the Funding section to read consistently as: “This study was supported by the Science and Technology Program of Henan Province [Grant No. 232400410004].”

**Comment 4 (Role of Funder):** Please state what role the funders took in the study.

**Response:** We have added the following statement in the Cover Letter and Funding section: “The funders had no role in study design, data collection and analysis, decision to publish, or preparation of the manuscript.”

**Comment 5 (Table references):** Please ensure that Table 1, 2, 3, and 7 are cited in the text.

**Response:** We appreciate the reminder. All tables are now properly cited in the main text, and numbering consistency has been verified.

**Responses to Reviewer #1**

**Comment 1:** Model performance and overfitting (Table 4, line 195).

“The results suggest serious overfitting. Please use repeated nested cross-validation (RNCV) and permutation tests to validate performance.”

**Response:** We appreciate the reviewer’s insightful suggestion. To verify that the reported results are statistically robust and not affected by overfitting, we have added two complementary validation analyses while keeping the original modeling framework unchanged (FE-OLS + XGBoost residuals).

(1) Repeated Nested Cross-Validation (RNCV):

We implemented a 5-fold × 10-repeat RNCV, grouping samples by year to respect the temporal structure of the panel data. The outer loop estimates generalization performance, while the inner loop (3-fold) selects the regularization strength for the XGBoost residual learner. The results show stable performance across all folds (FE-OLS: RMSE = 0.0318 ± 0.0137, R² = 0.9845 ± 0.0169; FE + XGB: RMSE = 0.0289 ± 0.0127, R² = 0.9873 ± 0.0146), indicating good generalization and no overfitting.

(2) Permutation Test:

We further conducted a 500-permutation test by randomly shuffling the training outcomes while keeping the feature matrix fixed. The observed test-set R^2^(0.924) was far higher than the null-distribution mean (−3.02 ± 2.06), yielding a right-tailed p = 0.002. This confirms that the model’s predictive power is highly unlikely to have occurred by chance.

The detailed results are provided in S3 and S4 Tables, which report the mean ± SD performance metrics (RMSE, MAE, R²) and the permutation-test significance results, respectively.

These additions demonstrate that the hybrid FE + XGB model maintains stable and statistically significant predictive performance, thereby addressing the reviewer’s concern regarding overfitting.

**Comment 2:** Data treatment (line 195).

“Please explain why linear interpolation was used and justify over alternatives.”

**Response:** We appreciate the reviewer’s insightful comment. In the revised manuscript, we have clarified the rationale for using linear interpolation for missing data imputation.

Specifically, because the dataset is a balanced panel covering annual observations for 17 prefecture-level cities from 2010–2023, the missing values mainly occur sporadically within continuous time series. Linear interpolation was therefore chosen as it preserves the temporal continuity and monotonic trends of each city’s fiscal and income variables while avoiding distortions that may arise from mean or regression-based imputation.

A clarifying sentence has been added to the Data treatment and quality checks section (line 120).

**Comment 3:** Multicollinearity (line 201).

“Please provide a table of VIF values.”

**Response:** We thank the reviewer for this valuable suggestion. We have now computed and reported the Variance Inflation Factor (VIF) for all explanatory variables used in the FE-OLS baseline regression. The results are presented in S1 Table , showing that all VIF values are below the conventional threshold of 10 (and mostly below 5), indicating that multicollinearity is not a serious concern in our model.

Accordingly, we have revised the manuscript to explicitly reference this table in the methodology section (line 128 in the original submission).

**Comment 4:** Hyperparameter tuning (line 230).

“Grid search is mentioned but results are not reported.”

**Response:** We thank the reviewer for this helpful suggestion. We have now included the detailed results of the hyperparameter tuning for the XGBoost residual learner.

As reported in S2 Table (Hyperparameter Tuning), the optimal parameter combination obtained from 5-fold time-series cross-validation was {colsample_bytree = 0.6, learning_rate = 0.1, max_depth = 4, n_estimators = 400, subsample = 1.0}, achieving the lowest CV RMSE = 0.0159.

This table has been added to the Appendix to ensure full transparency and reproducibility.

**Comment 5:** SHAP interpretation.

“Clarify that SHAP reflects model importance, not causality.”

**Response:** We thank the reviewer for this important clarification. We fully agree that SHAP values explain the model’s internal feature attributions rather than causal effects in the real world.

In the revised manuscript, we have added a sentence in the SHAP interpretation section to explicitly state this distinction:

“It should be noted that SHAP values quantify each feature’s contribution to the model’s predicted outcome, reflecting associations captured by the fitted model rather than causal relationships in reality.”

This clarification has been incorporated in the SHAP Explainability subsection (line 183 in the revised manuscript).

**Responses to Reviewer #2**

**Comment 1**: Integration of Sections 1–3.

“Please combine the introduction, theory, and literature review into one cohesive Introduction.”

**Response:** We appreciate the reviewer’s insightful suggestion regarding the structure of the introductory sections. In the revised manuscript, the former Sections 1 (Introduction), 2 (Theoretical framework), and 3 (Literature review) have been merged into a single, integrated “Introduction” section to improve narrative flow and logical coherence.

This unified section now weaves prior empirical findings directly into the discussion of theoretical mechanisms. For instance, the revised introduction links the “productive–unproductive expenditure” theory (Devarajan et al., 1996) and the “threshold crowding-out hypothesis” (Hansen, 1999; Lu, 2023) with recent empirical evidence on diminishing fiscal returns from Chinese provincial data (Li & Liu, 2021; Yuan & Wang, 2024).

Moreover, the new version explicitly identifies the methodological gap—that previous studies relied on linear or parametric threshold models that could not detect higher-order interactions among fiscal items—thereby motivating our SHAP-informed machine learning framework.

Overall, this restructuring produces a more cohesive and conceptually integrated introduction, clarifying both the theoretical foundation and the novelty of our approach.

**Comment 2:** Comparison section.

“The discussion is too descriptive; provide deeper critical analysis.”

**Response:** We thank the reviewer for this constructive comment. The Discussion (Comparison) section has been completely rewritten to enhance analytical depth and reduce descriptive listing. The revised version now explicitly contrasts our findings with prior literature and explains the mechanisms behind convergence or divergence.

Specifically, we now:

Interpret the inverted-U patterns for education and health spending as empirical confirmation of the Armey curve within a Chinese local-fiscal context, extending the results of Rijkers & Söderbom (2020) and refining Chen (2019)’s linear findings.

Clarify that our hybrid FE + XGBoost model resolves inconsistencies in earlier studies that failed to detect non-linear turning points due to functional-form constraints.

Introduce a new synthesis paragraph explaining how the machine-learning approach reconciles conflicting empirical evidence by modelling multi-item fiscal interactions and identifying stable expenditure thresholds.

Add recent empirical references (e.g., Economic Systems, 2025; SAGE Open, 2024) to situate our contribution within the newest research stream on explainable ML in development economics.

This restructured discussion transforms the section from a descriptive summary into a critical and integrative analysis that positions our results as both confirmatory and corrective within the existing literature.

**Comment 3:** Number of figures.

“Reduce figures, combine or convert some to tables.”

**Response:** We thank the reviewer for this helpful observation. In the revised manuscript, we have carefully streamlined and consolidated the figure set to improve clarity and interpretability. Specifically, the number of figures has been reduced from eleven to four by merging related visual elements into multi-panel layouts:

1. New Figure 1 (a–d) now combines the original Figures 1–3 and the permutation-test figure, presenting all model validation results—including test-set performance, cross-validation, permutation significance, and predicted-vs-actual plots—within a single, coherent framework.
2. New Figure 2 (a–d) integrates the global SHAP summary and feature-importance results that were previously shown separately (old Figs. 4–6), allowing readers to compare global contributions and interactions in one view.
3. Figures that lacked standalone interpretive value (such as the former Fig. 11 on sensitivity analysis) have been removed, and the corresponding information is now reported in Table 7 (Robustness Summary).

These revisions significantly improve readability and reduce redundancy while preserving all essential analytical content. The total number of figures has been cut by more than half, and all quantitative details are now clearly documented either in the main tables or in the Appendix.

Closing Statement

We sincerely thank the editor and reviewers again for their valuable feedback, which has significantly improved the manuscript in terms of rigor, clarity, and policy relevance. We hope the revised version meets the standards of PLOS ONE and look forward to your positive consideration.

Sincerely,

Jinshuai Zhang, Tingting Zhang
