# Supplementary material for: Non-linear impacts of local fiscal expenditure on farmers’ income: A SHAP-informed machine-learning analysis
Source: PLoS One. 2026 Mar 16;21(3):e0340008. doi: 10.1371/journal.pone.0340008 (PMC12991217; doi:10.1371/journal.pone.0340008)
Supplement: Attachment — Submitted filename: Response_to_Reviewers_auresp_2.docx [file pone.0340008.s008.docx]

Manuscript ID: PONE-D-25-30509R1

Title: Non-linear impacts of local fiscal expenditure on farmers’ income: A SHAP-informed machine learning analysis

Dear Editor and Reviewers,

We would like to express our sincere gratitude to the Academic Editor and reviewers for their constructive and thoughtful comments. We have carefully revised the manuscript according to all suggestions. Below, we provide a detailed, point-by-point response to each comment. All changes in the manuscript are highlighted in the “Revised Manuscript with Track Changes”.

**Response to Reviewer #1**

Comment 1: Model overfitting remains a concern. The reviewer acknowledges RNCV and permutation testing, but the high training R² (0.9996) vs. test R² (0.9245) suggests lingering overfitting. This issue must be explicitly addressed.

**Response:**

Thank you very much for raising this important point. We agree that the near-perfect in-sample R² requires clear explanation. We have now added a dedicated discussion in both the Methodology and Results/Discussion sections to clarify why this phenomenon is inherent to the residual-learning framework rather than evidence of harmful overfitting.

(1) Methodology – structural explanation added

We now explain that the second-stage learner is trained on fixed-effects residuals, whose variance is substantially compressed after city and year fixed effects absorb most systematic variation. As a result, tree-based learners can exhibit very high in-sample fit due to the low-variance target, even in the absence of noise memorization. This explanation has been added to the Methodology section (Lines 174-184).

(2) Results – overfitting diagnostics added and expanded

We expanded the Results section by presenting a more explicit set of overfitting diagnostics (Lines 239-241). Repeated nested cross-validation, rolling forecast-origin validation, and permutation-based null benchmarking all show that the FE + XGBoost model generalizes well. The cross-validated R² values are closely aligned with the test-set R², the rolling validation exhibits stable temporal performance, and the permutation test confirms that the observed predictive accuracy cannot arise by chance. A new concluding sentence has been added to state clearly that these diagnostic results jointly demonstrate the absence of harmful overfitting.

(3) Discussion – interpretive clarification added

We added a paragraph clarifying that the high in-sample R² is an inherent property of variance-compressed residual learning and that the empirical nonlinearities are robust across diagnostic tests. This provides conceptual closure and improves interpretability (Lines 475-483).

Together, these revisions directly address the reviewer’s concern and make the modeling logic clearer, more transparent, and more statistically rigorous. Thank you again for highlighting this issue — the added explanation improves the transparency and robustness of our modelling strategy.

**Response to Reviewer #2**

**Comment 1:** Numerical values in some tables (e.g., Tables 5 and 7) contain too many decimal places, reducing readability. Please round values to two or three decimals and unify formatting.

**Response:**

Thank you very much for your helpful observation regarding the numerical precision and formatting of the tables.

Following your suggestion, we conducted a thorough revision of all tables in the manuscript and standardised the decimal places according to the characteristics and statistical meaning of each variable type. For instance, model evaluation metrics such as RMSE, MAE, and R² are now reported with four decimal places to preserve the distinction between competing models with very small error magnitudes. Fiscal expenditure thresholds expressed in yuan are presented with two decimal places, which avoids unnecessary precision while maintaining interpretive clarity. SHAP-derived turning points in logarithmic form are also consistently shown with two decimal places, consistent with their estimation accuracy. All such adjustments have been clearly reflected and marked in the revised tables. In addition, to further enhance readability and align the manuscript with academic formatting standards, all tables have been reformatted in accordance with the formatting specifications of PLOS ONE, ensuring that their structure is fully consistent with the journal’s requirements.

These revisions significantly improve clarity, visual consistency, and the professional presentation of the empirical results. We sincerely appreciate your attention to these details.

**Comment 2:** The manuscript subheadings—particularly in the Discussion—are too many and too similar in size. Consolidation is recommended.

**Response:**

Thank you very much for your helpful suggestion regarding the structure of the Discussion section. We agree that the previous version included too many internal sub-headings and enumerated items, which made the section appear fragmented and less coherent. In response, we have substantially streamlined the Discussion by retaining only the three main subsections—*Comparison with Prior Studies*, *Theoretical & Practical Implications*, and *Limitations & Future Research*—and removing all lower-level sub-headings within these sections. The content previously organised under headings such as “Model interpretability and reliability,” “Theoretical refinement,” “Policy implications,” and “Methodological insight” has now been integrated into unified narrative paragraphs to improve flow and readability. These revisions make the Discussion more cohesive, stylistically consistent with journal expectations, and easier for readers to follow.

We sincerely appreciate the reviewer’s constructive suggestion, which has helped improve the clarity and presentation quality of the manuscript.

We sincerely thank both reviewers again for their thoughtful and constructive feedback. All suggested revisions have been incorporated, and we believe the manuscript is now significantly improved in clarity, rigor, and presentation.

Thank you very much for your time and effort.

Sincerely,

Tingting Zhang, Jinshuai Zhang

Anyang Institute of Technology

Email: ztt_ag@163.com
